# Elevated peripheral blood lymphocyte-to-monocyte ratio predicts a favorable prognosis in the patients with metastatic nasopharyngeal carcinoma

**DOI:** 10.1186/s40880-015-0025-7

**Published:** 2015-06-10

**Authors:** Rou Jiang, Xiu-Yu Cai, Zhong-Han Yang, Yue Yan, Xiong Zou, Ling Guo, Rui Sun, Dong-Hua Luo, Qiu-Yan Chen, Pei-Yu Huang, Yan-Qun Xiang, Xing Lu, Lin Wang, Wei-Xiong Xia, Hai-Qiang Mai, Ming-Yuan Chen

**Affiliations:** Sun Yat-sen University Cancer Center; State Key Laboratory of Oncology in South China; Collaborative Innovation Center for Cancer Medicine, Guangzhou, Guangdong 510060 P. R. China; Department of Nasopharyngeal Carcinoma, Sun Yat-sen University Cancer Center, Guangzhou, Guangdong 510060 P. R. China; Department of Cancer Prevention, Guangzhou, Guangdong 510060 P. R. China; Department of Medical Oncology, Sun Yat-sen University Cancer Center, Guangzhou, Guangdong 510060 P. R. China; Department of Biochemistry, Zhongshan School of Medicine, Sun Yat-sen University, Guangzhou, Guangdong 510080 P. R. China

**Keywords:** Metastatic nasopharyngeal carcinoma, Lymphocyte count, Monocyte count, Lymphocyte-to-monocyte ratio, Overall survival, Prognosis

## Abstract

**Introduction:**

Patients with metastatic nasopharyngeal carcinoma (NPC) have variable survival outcomes. We have previously shown that an elevated peripheral blood lymphocyte-to-monocyte ratio (LMR) is associated with an increased metastatic risk in patients with primary NPC. The present study aimed to investigate the prognostic value of pretreatment LMR in a large cohort of metastatic NPC patients.

**Methods:**

Clinical data of 672 patients with metastatic NPC diagnosed between January 2003 and December 2009 were analyzed. The peripheral lymphocyte and monocyte counts were retrieved, and LMR was calculated. Receiver operating characteristic (ROC) curve analysis and univariate and multivariate COX proportional hazards analyses were performed to evaluate the association of LMR with overall survival (OS).

**Results:**

Univariate analysis revealed that an elevated absolute lymphocyte count (≥1.390 × 10^9^/L) and LMR (≥2.475) as well as a decreased monocyte count (<0.665 × 10^9^/L) were significantly associated with prolonged OS. Multivariate Cox proportional hazard analysis showed that LMR (hazard ratio [HR] = 0.50, 95 % confidence interval [CI] = 0.41–0.60, *P* < 0.001), absolute lymphocyte count (HR = 0.77, 95 % CI = 0.64–0.93, *P* = 0.007), and monocyte count (HR = 1.98, 95 % CI = 1.63–2.41, *P* < 0.001) were independent prognostic factors. By stratification analyses, only LMR remained a significant predictor of prognosis.

**Conclusion:**

We identified pretreatment LMR as an independent prognostic factor for patients with metastatic NPC. Independent validation of our findings is needed.

## Background

Nasopharyngeal carcinoma (NPC) is a squamous cell carcinoma that occurs in the epithelial lining of the nasopharynx, with high incidence recorded in South China and Southeast Asia [1, 2]. With the increasing application of high-precision radiotherapy, distant failure is expected to become a predominant cause of death from NPC [3, 4]. Once metastasis is diagnosed, the overall survival (OS) of patients is typically under 15 months with palliative chemotherapy. Nevertheless, retrospective studies have shown great differences in the survival outcomes in patients with variable affected anatomic sites and different numbers of metastases [5, 6]. For specific subgroups, OS may exceed 10 years [6]. Therefore, a valuable marker to predict prognosis is desirable to facilitate individualized treatments and thus to achieve better outcomes for patients with metastatic NPC.

In the last decade, pretreatment peripheral differential leukocytes, such as lymphocytes and monocytes, have been found to be associated with prognosis in various cancers. A high pretreatment lymphocyte count has been determined to be associated with the good prognosis of patients with acute lymphoblastic leukemia [7], metastatic gastric cancer [8], and NPC [9]. A high monocyte count has been found to be a poor independent prognostic factor in patients with diffuse large B-cell lymphoma and metastatic melanoma [10]. An elevated lymphocyte-to-monocyte ratio (LMR) has been reported to be a prognostic factor for clinical outcome in patients with diffuse large B-cell lymphoma and Hodgkin’s lymphoma [11]. Recently, we have shown that an elevated LMR is associated with an increased metastatic risk in patients with primary NPC [12]. However, there have been few studies of the prognostic value of LMR in patients with metastatic NPC. Therefore, the current study was designed to analyze the effect of pretreatment LMR on OS in these patients.

## Patients and Methods

### Patient selection and data collection

Clinical data of patients with metastatic NPC referred to Sun Yat-sen University Cancer Center (SYSUCC) between January 2003 and December 2009 were reviewed. All of the included patients met the following criteria: 1) pathologically confirmed World Health Organization (WHO) type II or III NPC; 2) radiographically detectable metastatic disease; 3) a Karnofsky Performance Status score of ≥70; and 4) available clinical information and laboratory data at the diagnosis of metastasis. The exclusion criteria were as follows: 1) patients with a self-reported acute infection or hematologic disorder and 2) those with another type of malignancy. The Union for International Cancer Control/American Joint Committee on Cancer (UICC/AJCC) TNM classification system (6th edition, 2002) was used for staging. This study protocol was approved by the Clinical Ethics Review Board of SYSUCC.

As part of the physical examination, peripheral blood was collected before treatment, and both peripheral lymphocytes and monocytes were counted using a Sysmex XE-5000 automated hematology analyzer (Sysmex, Kobe, Japan). The peripheral LMR was calculated as the ratio of the absolute peripheral lymphocyte count to monocyte count. The serum antibody titers of Epstein-Barr virus (EBV) immunoglobulin A against virus capsid antigen (VCA/IgA) and early antigen (EA/IgA) were detected by enzyme-linked immunosorbent assay [13].

### Treatment and follow-up

According to our institutional guidelines for the palliative treatment of metastatic NPC, cisplatin-based systemic chemotherapy was provided to all patients as a basic treatment. Definitive radiotherapy targeting both the primary tumor and its regional lymph nodes (locoregional radiotherapy, lrRT) was administered to the patients with metastasis at presentation for local symptomatic relief or as a part of a multidisciplinary approach, as previously described [14–16]. The evaluation of tumor response to therapy was based on a computed tomography (CT) or magnetic resonance imaging (MRI) scan. After the treatment was completed, the patients were evaluated at 3-month intervals for the first 3 years and every 6 months thereafter or until death. The last follow-up date was December 31, 2013 for all available patients.

### Statistical analysis

Statistical analyses were performed using SPSS software (version 16.0, SPSS Inc., Chicago, IL, USA). OS was defined as the period between the first diagnosis of metastatic NPC and death or the last follow-up. The receiver operating characteristic (ROC) curve analysis was performed to select the most appropriate cut-off points for absolute lymphocyte and monocyte counts as well as LMR to stratify the patients at high risk of malignancy-related death. Univariate and multivariate analyses of clinicopathologic variables were performed using Cox proportional hazards regression models. Actuarial OS was plotted against time using the Kaplan–Meier method, and differences between the survival curves were assessed using the log-rank test. The correlation of LMR with different clinicopathologic characteristics was evaluated by Spearman’s rank correlation coefficient (*r*). The chi-square test was used to analyze differences in proportions. A two-sided *P* < 0.05 was considered significant.

## Results

### Patients’ characteristics

In total, the data for 672 patients were retrievable. The baseline patient characteristics are shown in Table 1. The median age at the time of diagnosis was 46 years (range, 13–79 years), and 546 (81.2 %) patients were male. Two hundred and ninety-seven (44.2 %) patients had distant metastases at presentation. The bones (58.3 %) were the most common site of metastasis, followed by the liver (34.1 %), lung (31.2 %), and extraregional lymph nodes (26.0 %). Most of the patients (84.4 %) had metastases at multiple sites at the time of diagnosis. By the last follow-up, 458 (68.2 %) of the 672 patients died. The 1-, 2-, and 3-year OS rates for the entire patient cohort were 81.1 %, 52.5 %, and 37.2 %, respectively.

**Table 1:** Clinicopathologic characteristics of the 672 patients with metastatic nasopharyngeal carcinoma (NPC)

Characteristic	Total (cases)	Deaths [cases (%)]	Unadjusted HR (95 % CI)	*P* value
Total	672	458		
Gender				
Male	546	371 (67.9)	Reference	0.700
Female	126	87 (69.0)	1.05 (0.83, 1.32)	
Age (years)				
<46	316	213 (67.4)	Reference	0.124
≥46	356	245 (68.8)	1.16 (0.96, 1.39)	
T stage*				
T1-2	273	184 (67.4)	Reference	0.975
T3-4	399	274 (68.7)	1.00 (0.83, 1.20)	
N stage*				
N0-1	273	172 (63.0)	Reference	<0.001
N2-3	399	286 (71.7)	1.42 (1.18, 1.72)	
Metastasis at presentation				
Absent	375	261 (69.6)	Reference	0.825
Present	297	197 (66.3)	0.98 (0.81, 1.18)	
Number of metastatic lesions				
1	105	51 (48.6)	Reference	<0.001
≥2	567	407 (71.8)	2.06 (1.54, 2.77)	
Bone metastasis				
Absent	280	196 (70.0)	Reference	0.862
Present	392	262 (66.8)	1.02 (0.85, 1.22)	
Liver metastasis				
Absent	443	283 (63.9)	Reference	<0.001
Present	229	175 (76.4)	1.62 (1.34, 1,95)	
Lung metastasis				
Absent	462	309 (66.9)	Reference	0.847
Present	210	149 (71.0)	0.98 (0.81, 1.19)	
Extraregional lymph node metastasis				
Absent	497	328 (66.0)	Reference	0.060
Present	175	130 (74.3)	1.22 (0.99, 1.49)	
EBV VCA/IgA				
<1:80	80	48 (60.0)	Reference	0.413
1:80–1:320	395	270 (68.4)	1.15 (0.84, 1.56)	
≥1:640	197	140 (71.1)	1.17 (0.85, 1.63)	
EBV EA/IgA				
<1:10	128	84 (65.6)	Reference	0.385
1:10–1:20	216	149 (69.0)	0.97 (0.74, 1.27)	
≥1:40	328	225 (68.6)	0.91 (0.71, 1.16)	
Lymphocyte count (× 10^9^/L)				
<1.390	340	250 (73.5)	Reference	0.002
≥1.390	332	208 (62.7)	0.75 (0.63, 0.90)	
Monocyte count (× 10^9^/L)				
<0.665	428	263 (61.4)	Reference	<0.001
≥0.665	244	195 (80.0)	2.17 (1.80,2.62)	
LMR				
<2.475	335	269 (80.3)	Reference	<0.001
≥2.475	337	189 (56.1)	0.45 (0.37, 0.54)	

HR, hazard ratio; CI, confidence interval; EBV, Epstein-Barr virus; VCA/IgA, immunoglobulin A against viral capsid antigen; EA/IgA, immunoglobulin A against early antigen; LMR, lymphocyte-to-monocyte ratio. *The American Joint Committee on Cancer (AJCC) 2002 system was used for staging at the diagnosis of NPC

**Fig. 1:**
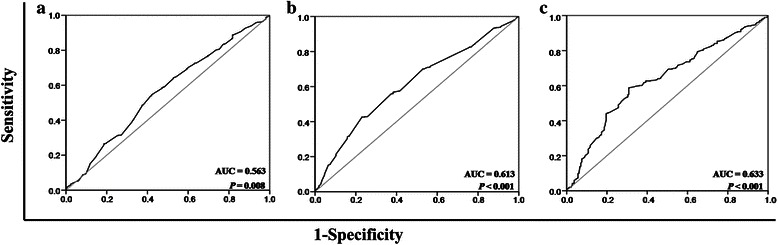
Receiver operating characteristic (ROC) curve analyses of cut-off points of baseline absolute lymphocyte and monocyte counts and lymphocyte-to-monocyte ratio (LMR) for overall survival (OS) analyses among patients with metastatic nasopharyngeal carcinoma (NPC). The cut-off points of the lymphocyte count (**a**), monocyte count (**b**), and LMR (**c**) for OS analyses were 1.390 × 10^9^/L, 0.665 × 10^9^/L, and 2.475, respectively. AUC, area under the ROC curve

The mean lymphocyte and monocyte counts were 1.462 × 10^9^/L (range, 0.100–5.850 × 10^9^/L) and 0.607 × 10^9^/L (range, 0.020–2.300 × 10^9^/L), respectively. The mean LMR was 3.219 (range, 0.180–69.000). The cut-off points of the absolute lymphocyte count, monocyte count, and LMR for OS as determined by ROC curve analyses were 1.390 × 10^9^/L, 0.665 × 10^9^/L, and 2.475, respectively (Fig. 1). A low LMR (<2.475) was significantly correlated with metastasis after radical therapy (*r* = 0.306, *P* < 0.001), involvement of more than one metastatic site (*r* = −0.150, *P* < 0.001), liver metastasis (*r* = −0.150, *P* < 0.001), and extraregional lymph node metastasis (*r* = −0.100, *P* = 0.009) (Table 2).

**Table 2:** Correlation of baseline LMR with clinicopathologic characteristics of patients with metastatic NPC

Characteristic	LMR < 2.475	LMR ≥ 2.475	*r* value	*P* value
Total	335	337		
Gender				
Male	277	269	0.037	0.342
Female	58	68		
Age (years)				
<46	157	159	−0.003	0.935
≥46	178	178		
T stage				
T1-T2	136	137	<0.001	0.988
T3-T4	199	200		
N stage				
N0-N1	138	135	0.012	0.765
N2-N3	197	202		
Metastasis at presentation				
Absent	238	137	0.306	<0.001
Present	97	200		
Number of metastatic lesions				
1	34	71	−0.150	<0.001
≥2	301	266		
Bone metastasis				
Absent	139	141	−0.004	0.927
Present	196	196		
Liver metastasis				
Absent	197	246	−0.150	<0.001
Present	138	91		
Lung metastasis				
Absent	224	238	−0.041	0.294
Present	111	99		
Extraregional lymph node metastasis				
Absent	233	264	−0.100	0.009
Present	102	73		
EBV VCA/IgA				
<1:80	36	44	−0.037	0.339
1:80–1:320	197	198		
≥1:640	102	95		
EBV EA/IgA				
<1:10	56	72	−0.055	0.151
1:10–1:20	109	107		
≥1:40	170	158		

Footnotes as in Table 1

### Univariate Cox proportional hazards regression analysis of clinicopathologic characteristics

**Fig. 2:**
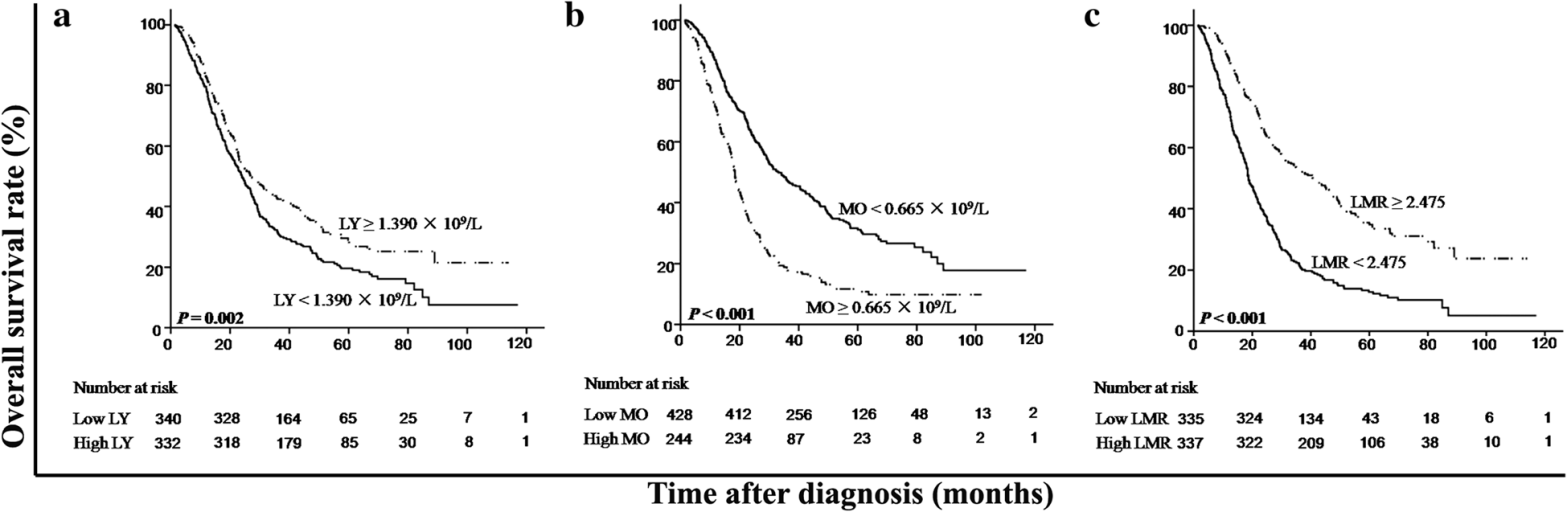
Kaplan–Meier overall survival (OS) analysis for patients with metastatic NPC. **a**, the OS rate was higher in the patients with a high absolute lymphocyte count than in those with a low count (*P* = 0.002). **b**, the OS rate was lower in the patients with a high absolute monocyte count than in those with a low count (*P* < 0.001). **c**, the OS rate was higher in the patients with a high LMR than in those with a low LMR (*P* < 0.001). LY, lymphocyte; MO, monocyte

As shown in Table 1, the absolute lymphocyte count, monocyte count, LMR, and other clinicopathologic characteristics (including age, gender, T stage, N stage, metastasis at presentation, number of metastatic lesions, metastasis sites [i.e., the bones, liver, lungs, and extraregional lymph nodes], and EBV-related antibodies [i.e., serum VCA/IgA and IgA/EA antibodies]) were subjected to univariate analysis. A higher absolute lymphocyte count, lower absolute monocyte count, and higher LMR were associated with longer OS (Fig. 2). Other variables, including N stage (hazard ratio [HR] = 1.42, 95 % confidence interval [CI] = 1.18-1.72, *P* < 0.001), number of metastatic lesions (HR = 2.06, 95 % CI = 1.54-2.77, *P* < 0.001), and liver metastasis (HR = 1.62, 95 % CI = 1.34-1.95, *P* < 0.001), were also found to be associated with OS (Table 1).

### Multivariate Cox proportional hazards regression analysis of clinicopathologic characteristics

**Fig. 3:**
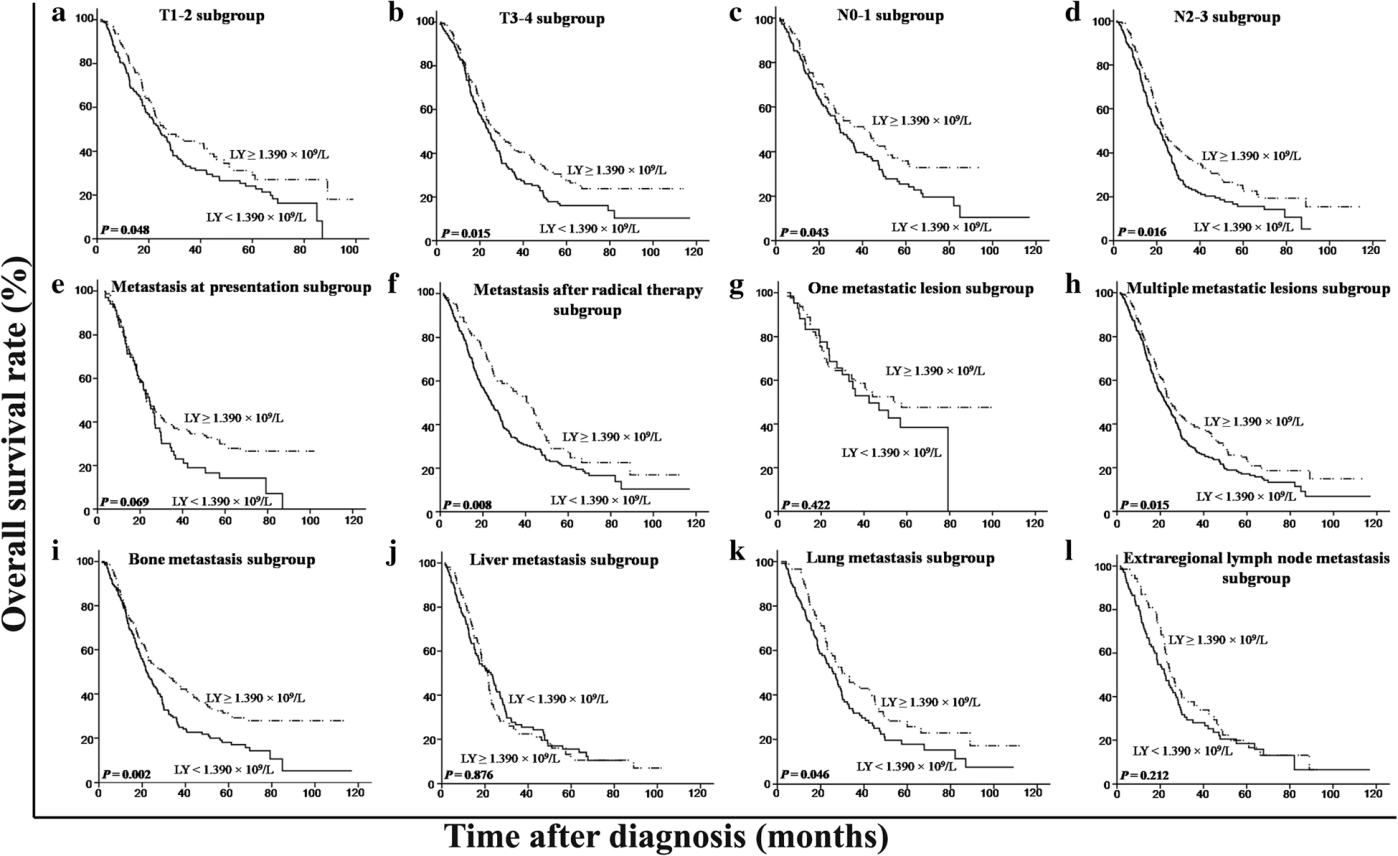
Kaplan-Meier OS analysis according to baseline absolute lymphocyte count in patients with metastatic NPC. In the T1-2 subgroup (**a**), the T3-4 subgroup (**b**), the N0-1 subgroup (**c**), the N2-3 subgroup (**d**), the subgroup with metastasis after radical therapy (**f**), the subgroup with multiple metastasis lesions (**h**), the bone metastasis subgroup (**i**), and the lung metastasis subgroup (**k**), the OS rates are higher in the patients with a high absolute lymphocyte count than in those with a low count (all *P* < 0.05). In the subgroup with metastasis at presentation (**e**), the subgroup with one metastasis lesion (**g**), the liver metastasis subgroup (**j**), and the extraregional lymph node metastasis subgroup (**l**), there is no significant difference between the two curves (all *P* > 0.05)

**Fig. 4:**
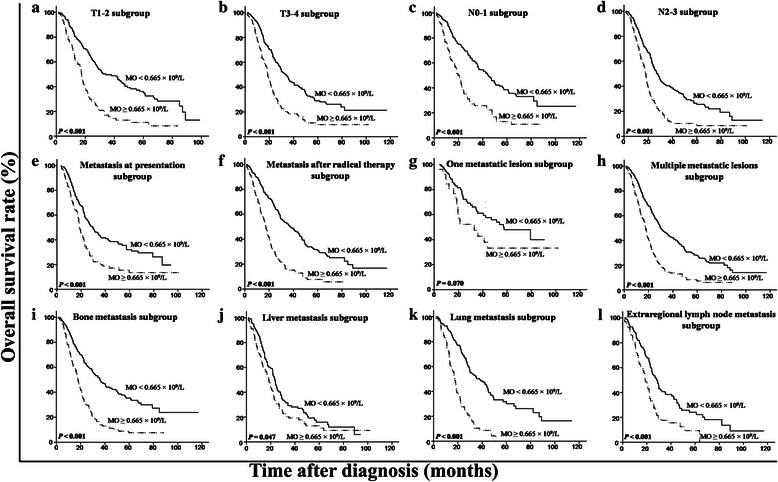
Kaplan-Meier OS analysis according to baseline absolute monocyte count in patients with metastatic NPC. In the T1-2 subgroup (**a**), the T3-4 subgroup (**b**), the subgroup with metastasis at presentation (**e**), the subgroup with multiple metastasis lesions (**h**), the bone metastasis subgroup (**i**), the liver metastasis subgroup (**j**), the lung metastasis subgroup (**k**), and the extraregional lymph node metastasis subgroup (**l**), the OS rates are lower in the patients with a high absolute monocyte count than in those with a low count (all *P* < 0.05). In the N0-1 subgroup (**c**), the N2-3 subgroup (**d**), and the subgroup with metastasis after radical therapy (**f**), the OS rate is higher in the patients with a high absolute monocyte count than in those with a low count (all *P* < 0.001). In the subgroup with one metastasis lesion (**g**), there is no significant difference between the two curves (*P* = 0.070)

**Fig. 5:**
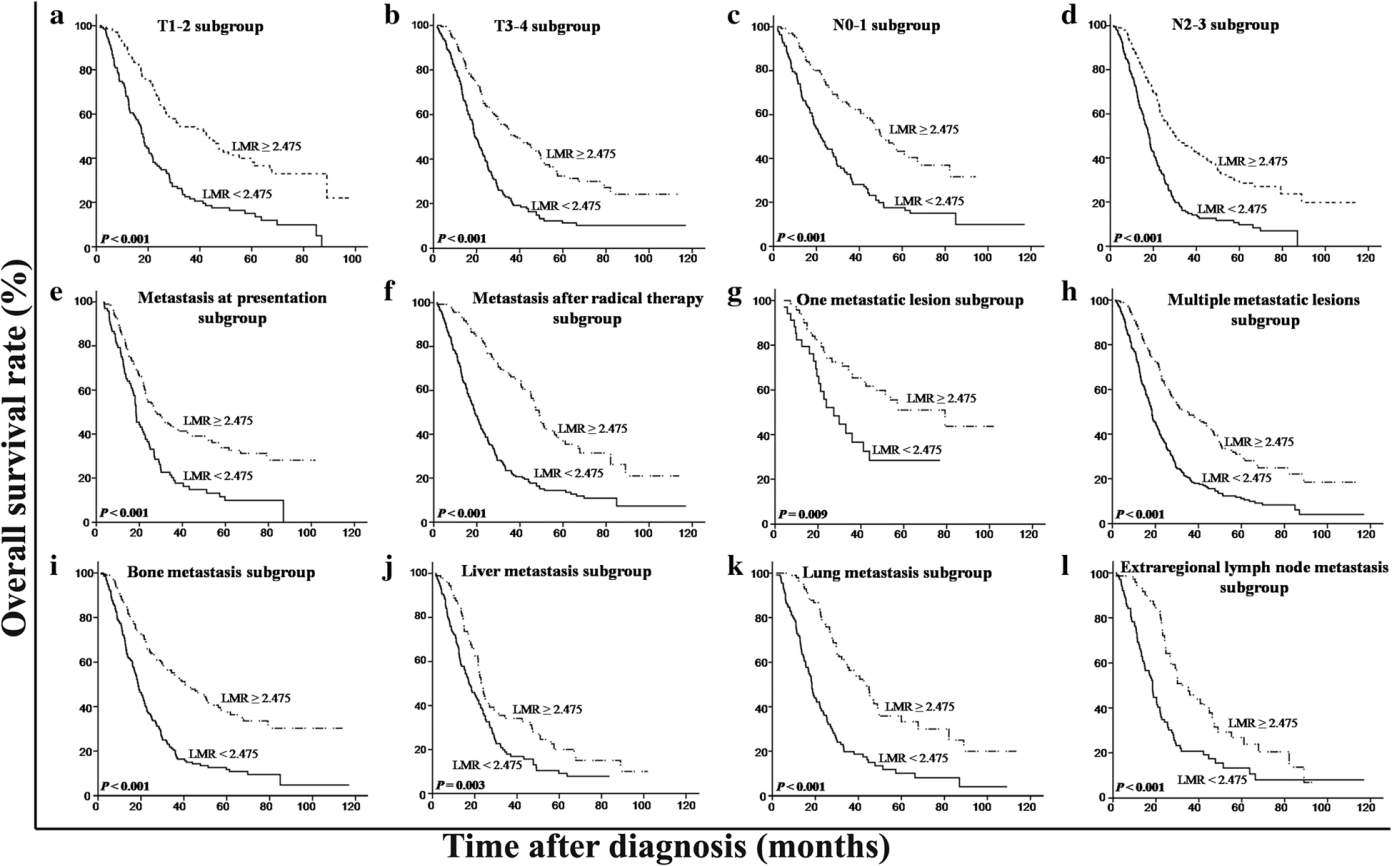
Kaplan-Meier OS analysis according to baseline LMR in patients with metastatic NPC. In the T1-2 subgroup (**a**), the T3-4 subgroup (**b**), the N0-1 subgroup (**c**), the N2-3 subgroup (**d**), the subgroup with metastasis at presentation (**e**), the subgroup with metastasis after radical therapy (**f**), the subgroup with one metastasis lesion (**g**), the subgroup with multiple metastasis lesions (**h**), the bone metastasis subgroup (**i**), the liver metastasis subgroup (**j**), the lung metastasis subgroup (**k**), and the extraregional lymph node metastasis subgroup (**l**), the OS rates are higher in the patients with a high LMR than in those with a low LMR (all *P* < 0.01)

We used a multivariate model to adjust for the confounders of the association of LMR with survival. The results showed that a high LMR was an independent predictor of a favorable OS (HR = 0.50, 95 % CI = 0.41–0.60, *P* < 0.001) (Table 3 Model 1, including LMR as a variable). In addition, both absolute lymphocyte and monocyte counts were analyzed for their independence from other covariates using the Cox model (Table 3 Model 2, including lymphocyte and monocyte counts as variables). LMR was not included here, considering the multicollinearity between LMR and absolute lymphocyte and monocyte counts. The results showed that the absolute lymphocyte count was an independent factor for a favorable prognosis (HR = 0.77, 95 % CI = 0.64–0.93, *P* = 0.007), whereas the absolute monocyte count was an independent inferior prognostic factor for patients with metastatic NPC (HR = 1.98, 95 % CI = 1.63–2.41, *P* <0.001) (Table 3 Model 2). After stratification by T stage, N stage, metastasis at presentation, metastasis after radical therapy, number of metastatic lesions, and metastatic sites, only LMR remained a significant predictor of prognosis (Figs. 3, 4 and 5). Moreover, an advanced N stage, the presence of two or more lesions, and liver metastasis were shown to be independent indicators of short OS (Table 3).

**Table 3:** Multivariate analysis of prognostic factors in patients with metastatic NPC

Characteristic	Model 1		Model 2	
	HR (95 % CI)	*P* value	HR (95 % CI)	*P* value
N stage (N2-3 vs. N0-1)	1.42(1.18, 1.72)	<0.001	1.39(1.15,1.68)	0.001
Number of metastatic lesions (≥2 vs. 1)	1.73(1.29, 2.32)	<0.001	1.87(1.40,2.52)	<0.001
Liver metastasis (present vs. absent)	1.38(1.14, 1.67)	0.001	1.29(1.06,1.57)	0.010
Lymphocyte count (≥1.390 × 10^9^/L vs. <1.390 × 10^9^/L)	NA	NA	0.77(0.64,0.93)	0.007
Monocyte count (≥0.665 × 10^9^/L vs. <0.665 × 10^9^/L)	NA	NA	1.98(1.63,2.41)	<0.001
LMR (≥2.475 vs. <2.475)	0.50(0.41, 0.60)	<0.001	NA	NA

NA, not applicable. Other footnotes as in Table 1

## Discussion

In the current study, we demonstrated that an elevated LMR was significantly associated with prolonged OS and was independent of the other variables assessed in predicting the prognosis of patients with metastatic NPC. Moreover, after stratification by T stage, N stage, metastasis at presentation, metastasis after radical therapy, number of metastatic lesions, and metastatic sites, LMR remained a significant predictor of prognosis.

There is substantial evidence in advanced cancer that the host systemic immune response is an important independent predictor of outcome and that pre-treatment measures of the systemic inflammatory immune response can be used to independently predict cancer patients’ survival [17]. Among many systemic inflammatory measures, the white blood cell (WBC) subset count (the neutrophil count [18] or the neutrophil-to-lymphocyte ratio [19]) is well known as an independent prognostic factor for survival [17]. However, evidence that LMR may have a prognostic role in cancer is limited. Recent reports have indicated that LMR was positively associated with survival outcomes in classical Hodgkin’s lymphoma [20], diffuse large B-cell lymphoma [21], metastatic non-small cell lung cancer [22], and NPC [12]. In the present study, we evaluated LMR as a prognostic indicator in 672 patients with metastatic NPC. Some of our results were consistent with previous findings. We found that an elevated LMR not only had a strong correlation with longer survival but also was an independent prognostic factor for survival, as determined by multivariate analysis using the Cox model. However, some of our results differed from those reported by Jin *et al.* [23], which have shown that the absolute lymphocyte count was not correlated with OS. In the current study, after adjusting for confounders, the absolute lymphocyte count remained as an independent prognostic factor for OS. The discordance between these two studies may be partially due to the different sample sizes: 672 patients were recruited in this study compared with 229 in the study by Jin *et al.* [23].

The mechanisms underlying the relationship between LMR and the prognosis of cancer patients remain unclear, which may be partially explained by the link between chronic inflammation and cancers [24–26]. It is a consensus that the adaptive immune system carries out immune surveillance and can eliminate newly formed tumors; however, effective adaptive immune responses are always suppressed in established tumors through several pathways, including the inhibition of dendritic cell differentiation and the activation and infiltration of regulatory T cells and tumor-associated macrophages [24]. Lymphocytes are crucial components of the adaptive immune system, and the presence of tumor-infiltrating lymphocytes has been reported to indicate the generation of an effective antitumor cellular immune response [27]. The peritumoral inflammatory response is thought to reflect the interaction between the tumor and the host. In previous studies, a high lymphocytic infiltrate has been linked with prolonged survival, independent of clinicopathologic characteristics, in breast cancer patients [28]. However, data supporting the association between intratumoral immune cells and blood-based cells constituting the systemic inflammatory response with OS are sparse. Previous studies have demonstrated an association between a low peripheral blood lymphocyte count and short survival in patients with different types of cancer [29, 30]. We have previously shown prolonged survival of primary NPC patients with elevated lymphocyte counts compared with those with decreased lymphocyte counts [12]. Monocytes are a subset of circulating white blood cells that can further differentiate into a range of tissue macrophages and dendritic cells [31]. It has been reported that monocytes secrete various proinflammatory cytokines, such as interleukin (IL)-1, IL-6, IL-10, and tumor necrosis factor-α (TNF-α), which have been associated with short survival and poor prognosis in patients with malignancy [32, 33]. Moreover, monocytes release monocyte chemo-attractant protein-1 (MCP-1) upon stimulation and mediate tumor-associated macrophage infiltration in solid tumors, which have been shown to produce a variety of chemokines, such as transforming growth factor-α (TGF-α), TNF-α, IL-1, and IL-6, to promote tumorigenesis, angiogenesis, and distant metastasis of malignant tumors [34, 35]. As a consequence, a high absolute monocyte count may indicate poor prognosis. Our findings also showed that a high monocyte count was significantly associated with short survival in patients with metastatic NPC.

LMR, which is defined as the absolute lymphocyte count divided by the absolute monocyte count, may reflect the diverse effects of monocytes and lymphocytes on tumor progression. Previous studies have demonstrated that normal human monocytes suppress either the phytohemagglutinin- or antigen-induced lymphocyte proliferative response when the monocyte-to-lymphocyte ratio is increased [36]. In the current study, although the lymphocyte count or monocyte count alone could predict the survival outcomes in patients with metastatic NPC, LMR outperformed them in this regard. Our results of multivariate Cox proportional hazard analysis showed that LMR and absolute lymphocyte and monocyte counts were independent prognostic factors. However, after stratification analysis, only LMR remained a significant predictor of prognosis. In addition, consistent with the findings of Pan *et al.* [37], we found that an advanced N stage, the presence of two or more metastatic lesions, and liver metastasis were independent prognostic factors for short OS, whereas lung or bone metastases were not associated with OS. We postulate that these findings may be associated with a unique biological behavior of NPC in the liver metastasis group.

### Conclusion

Our results show that LMR can function as an independent prognostic factor for patients with metastatic NPC. Moreover, this ratio can be easily determined with routine blood counts and is readily applicable clinically. We acknowledge that our findings are limited by the retrospective and single center nature of this study; thus, further independent validation of our findings is warranted.
